# Understanding of ‘generalist medical practice’ in South African medical schools

**DOI:** 10.4102/phcfm.v16i1.4324

**Published:** 2024-03-08

**Authors:** Langalibalele H. Mabuza, Mosa Moshabela

**Affiliations:** 1 Faculty of Health Sciences, Sefako Makgatho Health Sciences University, Pretoria, South Africa; 2 Faculty of Health Sciences, University of KwaZulu-Natal, Durban, South Africa

**Keywords:** generalist medical practice, preceptors, medical students, medical schools, South Africa

## Abstract

**Background:**

In South Africa, medical students are expected to have acquired a generalist competence in medical practice on completion of their training. However, what the students and their preceptors understand by ‘generalist medical practice’ has not been established in South African medical schools.

**Aim:**

This study aimed to explore what the students and their preceptors understood by ‘generalist medical practice’.

**Setting:**

Four South African medical schools: Sefako Makgatho Health Sciences University, University of KwaZulu-Natal, Walter Sisulu University and the University of the Witwatersrand.

**Methods:**

The exploratory descriptive qualitative design was used. Sixteen focus group discussions (FGDs) and 27 one-on-one interviews were conducted among students and their preceptors, respectively. Participants were recruited through purposive sampling. The inductive and deductive data analysis methods were used. The MAXQDA 2020 (Analytics Pro) software was used to arrange data, yielding 2179 data segments.

**Results:**

Ten themes were identified: (1) basic knowledge of medicine, (2) first point of contact with all patients regardless of their presenting problems, (3) broad field of common conditions prevalent in the community, (4) dealing with the undifferentiated patient without a diagnosis, (5) stabilising emergencies before referral, (6) continuity, (7) coordinated and (8) holistic patient care, necessitating nurturance of doctor–patient relationship, (9) health promotion and disease prevention, and (10) operating mainly in primary health care settings.

**Conclusion:**

The understanding of ‘generalist medical practice’ in accordance with internationally accepted principles augurs well in training undergraduate medical students on the subject. However, interdepartmental collaboration on the subject needs further exploration.

**Contribution:**

The study’s findings can be used as a guide upon which the students’ preceptors and their students can reflect during the training in generalist medical practice.

## Introduction

General medical practice has been described in various ways in literature, as ‘the broad preparation of a generalist, rather than a technically oriented specialist’,^1^ ‘the medical specialty that manages common and long-term illnesses in children and adults, focusing on overall health and well-being’,^2^ ‘generalist orientation’,^3^ ‘generalism’^4,5,6^ and ‘general practice’.^7^ The majority of literature pertaining to generalism is from the Western countries (Europe, the United States and Canada), where generalism is discussed in the context of postgraduate training, mainly family medicine.^8,9,10,11,12^ There is also evidence that students view general practice as the medical field of ‘low status within the medical hierarchy, … an easy default option … not considered to be a specialty’.^13^ These definitions paint a picture of generalist medical practice as the medical field with a broad focus on the management of common illnesses among all age groups in communities.

In 2002, the World Organisation of Family Doctors (WONCA) Europe outlined 11 characteristics of general practice: (1) the point of first medical contact within the healthcare system; (2) coordination of healthcare through collaboration with other professionals and maintaining patient advocacy; (3) adopting a person-centred approach, oriented to the individual, family and their community; (4) establishment of a practitioner–patient relationship over time; (5) provision of longitudinal continuity of care to meet the needs of the patient; (6) development of a decision-making process determined by the prevalence and incidence of community illnesses; (7) management of both acute and chronic health problems of patients; (8) management of a patient with an undifferentiated condition; (9) promotion of health and well-being through appropriate and effective intervention; (10) specific responsibility for the health of the community and (11) dealing with health problems in their bio-psycho-socio-cultural and existential dimensions.^9^

The authors of this article have combined the 11 characteristics into five main categories, as some address the same concept in healthcare delivery, for example, ‘Establishment of a practitioner-patient relationship over time’ (3), and ‘Provision of longitudinal continuity of care to meet the needs of the patient’ (4) both fall under the fourth category: ‘Provision of continuity of care’ (Table 1). Through these categories the authors intended to focus the study on its objectives in characterising ‘generalist medical practice’.

**TABLE 1:** Main characteristics of generalist practice.

Five main characteristics	Eleven characteristics
1. Dealing with an undifferentiatedpatient	The point of first medical contact within the healthcare system (1)
	Management of a patient with an undifferentiated condition (8)
2. Coordination of healthcare	Coordination of healthcare through collaboration with other professionals and maintaining patient advocacy (2)
3. Adopting a holistic patient care	Adopting a person-centred approach at the individual, family and community levels (3)
	Promotion of health and well-being through appropriate and effective intervention (9)
	Dealing with health problems in their bio-psycho-socio-cultural and existential dimensions (11)
4. Provision of continuity of care	Establishment of a practitioner–patient relationship over time (4)
	Provision of longitudinal continuity of care to meet the needs of the patient (5)
5. Dealing with a wide rangeof diseases	Development of a decision-making process determined by the prevalence and incidence of community illnesses (6)
	Management of both acute and chronic health problems of patients (7)
	Specific responsibility for the health of the community (10)

These categories of characteristics are derived from concepts of generalism discussed in the literature.^14,15^ Along these characteristics, this study aimed at exploring what medical students and their trainers understood by ‘generalist medical practice’.

In May 2021, a review of literature was conducted at the University of Toronto in Canada to assess the undergraduate (UG) education through a generalist lens using the Toronto – Generalism Assessment Tool (T-GAT).^16^ The tool assessed the inclusion of generalist principles in the medical training programmes and evaluated characteristic of generalist medical practice, including dealing with the undifferentiated patient, prevention and promotion, continuity and coordination of care, multidisciplinary team care (interprofessional collaboration) and patient advocacy. These characteristics bear similarity to those this study is focusing on. The assessment revealed an overall low inclusion of the generalist principles in student training, ranging from 10% to 30%.^16^ A conclusion was drawn that there was a paucity of generalist principles of medical education curricular materials in UG medical training in Canada. The use of this tool in South African medical schools would assist in determining the inclusion of generalist principles in the medical training programmes, as this has not been done thus far.

In South Africa, the training of UG medical students is conducted by various disciplines – specialists and subspecialists, as well as generalist practitioners. Specialists feature within the six major disciplines, which are: (1) General Surgery, (2) Internal Medicine, (3) Obstetrics and Gynaecology, (4) Paediatrics, (5) Family Medicine and (5) Psychiatry. The various categories of generalist practitioners are interns, community service doctors, medical officers, private practitioners (also called general practitioners – GPs) and family physicians. Family physicians are specialist generalist practitioners by virtue of their further training in the field of Family Medicine and Primary Health Care.^15^

According to chapter two of the Health Professions Council of South Africa (HPCSA): Regulations relating to the registration of students, UG curricula and professional examinations in medicine, ‘on successful completion of the curriculum’ a medical student ‘should have developed into a basic medical practitioner’.^17^ The ‘basic medical practitioner’ has been profiled as a student who has been trained in ‘an approved (South African) educational institution’ through a curriculum that conveyed ‘knowledge, skills, attitudes and appropriate modes of professional conduct’; prepared the student ‘for health promotion, the prevention or treatment of illness and rehabilitation of impairment’; equipped the student with ‘research and management abilities’ and ensured that the student bears ‘relevance to local health needs while satisfying international standards of excellence’.^17^ This profile entails attributes of generalist medical practice.^18^

The aim of this study was to explore the extent to which medical students and their preceptors in South African medical schools understood generalist medical practice within the international body of knowledge on the subject as outlined earlier, in addition to exploring peculiarities, if any, of their understanding within the South African healthcare context.

## Theoretical framework

The study used two theoretical frameworks, Vygotsky’s social constructivist theoretical framework (SCT) developed by Lev Vygotsky in 1934,^19^ and the situated learning theory (SLT), derived from the former by Lave and Wegner in 1991,^20^ as lenses through which the concept was studied. Both theories argue that in the learning setting, there are the ‘more knowledgeable others’ (experts) who train the ‘novices’ (apprentices) to also become experts in the community of practice, and that learning occurs best when it takes place in the context in which it is applied.^21^

Vygotsky’s theory takes a constructivist approach, whereby an individual is exposed to and participates in experiential learning to construct their reality.^22^ Through this lens, the study explored whether the students’ preceptors played their role as the ‘knowledgeable others’ to their students, by taking the students through the zone of proximal development (ZPD) towards understanding the concept of ‘generalist medical practice’, and also whether the training involved ‘scaffolding’ of knowledge and skills, whereby the students were given incremental clinical exposure and responsibility as they advanced through their years of training to attain understanding and independent practice capabilities.^23^

In addition to Vygotsky’s SCT, the study explored the students’ and their preceptors’ understanding of ‘generalist medical practice’, following Lave and Wenger’s SLT.^22^ This theoretical framework proposes that learning occurs in ‘communities of practice’ (CoP), through legitimate peripheral participation (LPP). The CoP entailed the students themselves, their preceptors and the training platforms where knowledge and skills impartation took place. Similar to Vygotsky’ SCT, Lave and Wenger’s LPP entailed the process by which a newcomer (in this case, a medical student) gradually works his or her way towards full participation in the CoP where there are the ‘more knowledgeable others’ (the students’ preceptors).^24^ According to this theory, ‘newcomers become members of a community initially by participating in simple and low-risk tasks that are nonetheless productive and necessary [*to*] further the goals of the community’.^22^ This study focused on the students in their clinical years of training, from fourth to sixth year. The understanding of the more senior students on the subject compared to their junior counterparts was explored to find out the extent to which the former had moved further towards ‘full participation’ in the CoP.^25^

Both theories assume a constructivist approach whereby the learner is given knowledge and skills enabling him or her to solve problems independently. Prior to implementation of the skills and knowledge for independent implementation of ‘generalist medical practice’, the students first needed to gain understanding of the concept, as imparted to them by their preceptors. The researchers in this study, in turn, needed to explore what the concept meant to the students and their preceptors, hence the choice of the exploratory descriptive qualitative (EDQ) study design in relation to the theoretical frameworks. In a similar manner, it became appropriate to conduct data collection by means of interviews: focus group discussions (FGDs) and in-depth interviews for the students and their preceptors, respectively, as these also followed the exploratory trend. Data interpretation through the lens of the theoretical frameworks has been elaborated on in the discussion section of this article.

Throughout the study, the theories gave insight into the researchers in exploring the understanding of the students and their preceptors regarding ‘generalist medical practice’ within the globally derived parameters of the concept, as already stated earlier.^14,15,18^

## Research methods and design

### Study design

This study used the EDQ study design.^26^ The researchers of this study found this design appropriate as it allowed the participants to contribute to the development of new knowledge (medical students and their preceptors’ understanding of ‘generalist medical practice’) in the training of UG medical students. At the time when this study was conducted, the researchers of this study had conducted a scoping study on the training of UG medical students on general medical practice and primary health care^25^ and did not find studies that reported on what medical students and their trainers understood by ‘generalist medical practice’. Most literature addressed some aspects of this subject at postgraduate medical training.^8,9,10^

This article focuses on one aspect of the principal researcher’s PhD project, which explored the training of medical students in generalist medical practice and primary health care (PHC) in South African medical schools. The study comprised four objectives: (1) what the UG medical students and their preceptors understood by ‘generalist medical practice’; (2) what the students and their preceptors understood by ‘primary health care’; (3) the experiences of the students and their preceptors regarding training in generalist medical practice and (4) the experiences of the students and their preceptors in primary health care training. This article reports on the first objective.

### Study setting

Four medical schools consented to participate in the study: Walter Sisulu University (WSU), Sefako Makgatho Health Sciences University (SMU) and the University of the Witwatersrand (WITS), including the University of KwaZulu-Natal (UKZN), which had secured a National Research Foundation (South Africa) grant. The UKZN had invited all universities with a medical school to a study to determine Transformation in Medical Education (TiME) in South Africa.^27^ Transformation in Medical Education aimed at transforming medical education in South Africa to be on par with global developments in medical education. The emphasis was to produce socially accountable fit-for-purpose healthcare practitioners for the country. In this multiparticipatory collaboration, the principal researcher of this study wants to explore the training of UG medical students in generalist medical practice and primary health care. To this end, this study reports on the exploration of the understanding of the medical students and their preceptors regarding ‘generalist medical practice’. It is addressing one of the four objectives as explained earlier.

Regarding the medical schools, each is situated in a geographical location representative of the country’s diverse geopolitical spectrum: WITS mainly urban, WSU mainly rural, SMU mainly urban with a rural component and UKZN mainly rural with an urban.^28^ Students and their trainers placed in different geopolitical environments have been found to encounter different geopolitical determinants of health and assume different approaches to meet those challenges.^29^ The researchers explored the understanding of ‘generalist medical practice’ by students and their preceptors in the varying geopolitical environments within South Africa. Although this diverse geopolitical spectrum provided the described institutional variability, their selection was convenient,^30^ rather than purposive,^31^ as it was based on their willingness to participate.

### Positionality

Regarding the principal researcher’s positionality, at the time of the study he was a family physician involved in the training of UG medical students in one of the four medical schools constituting the study setting. This position was declared to both the students and their preceptors during data collection. The principal researcher had no academic relationship with the participants in the other three medical schools.

Family Medicine, as a discipline, follows a generalist medical training approach among students,^32^ compared to specialist disciplines, which focus solely on their specialties; for example, obstetricians will confine themselves to the management of female patients pre- and peri-natally.^33^ During his training of the medical students, the principal researcher developed an interest in finding out how the various disciplines (including Family Medicine) trained students in ‘generalist medical practice’, and particularly, in this article what they understood by this concept in their respective disciplines.

For reflexivity, the main researcher remained conscious of this position during data collection, analysis and interpretation.^34^ Reflexivity has been defined as ‘the researcher’s ability to be able to self-consciously refer to him or herself in relation to the production of knowledge about research topics’.^35^ For example, as a family physician, the principal researcher interviewed fellow family physicians with whom he shared the same specialty background. He had to be self-aware that conclusions are not based on the assumptions emanating from this background. Reflexivity was enhanced through jotting down all comments made by the participants, the researcher’s thoughts that came during and after the interviews, and making reflective summaries soon after each interview had been conducted.

### Study population

The target population comprised the heads of departments (or their MBChB course coordinators) (Online Appendix 1) who were actively involved in the training of UG medical students in the major clinical disciplines mentioned in the introduction section earlier, as well as UG medical students in their clinical years of training (MBChB 4–6) (Online Appendix 2).

### Sampling strategy

The expert purposive sampling strategy was used for both the students and their preceptors.^31^ The researchers intentionally recruited the students who were undergoing the training in their respective years of study and the preceptors who were involved in the former’s training. These were information-rich participants regarding the subject under study.^36^ Student recruitment was performed through their class leadership who notified their colleagues and afforded the researchers the platform (physical or online) to contact the students. Recruitment of the students’ preceptors was arranged on a one-on-one basis per institution. Students’ FGDs were conducted in a lecture room of their choice, while the in-depth interviews with the students’ preceptors occurred in their offices. Twenty-seven in-depth interviews were conducted among the preceptors, while 16 FGDs, amounting to 102 students, were conducted at the four medical schools. Participation in this was voluntary.

Each FGD comprised five to eight students as recommended,^37^ who were in their clinical years (MBChB 4–6). In each institution, one homogeneous FGD was arranged per year group (MBChB 4, 5 and 6), and the fourth FGD was heterogeneous, comprising about two students from each year group. The latter was performed to ensure maximum variability among the student groups.^36^ Each in-depth interview and FGD lasted for an average of 1 h.

### Data collection

The principal researcher, assisted by a research assistant, collected data from 2016 to 2020 at the four medical schools: WSU 2016, WITS 2017–18, UKZN 2018–19 and SMU 2020. The research assistant was a 6-year medical student at the beginning of the study. He was being mentored by the principal researcher on research methodologies. His assistance ended when he qualified at the end of 2016. In this study, his main role was to take field notes and acquire students’ learning material from each medical school to corroborate information obtained through the interviews. The students’ guidelines varied in their curricular content per institution. However, that was not the focus of this study.

All interviews were conducted by the principal researcher. The exploratory question used to open discussions with each students’ preceptor was: ‘As a preceptor of undergraduate medical students, what is your understanding of generalist medical practice?’ An interview guide with prompts (Online Appendix 3) was used to focus the discussions on the study aim and objectives.

Regarding the FGDs conducted among the medical students, each comprised the recommended four to eight participants.^37^ The exploratory question to open discussions was: ‘As undergraduate medical students in this institution, what do you understand by “generalist medical practice”?’ Similar to their preceptors, there were also prompts for the students (Online Appendix 3). The duration of each FGD was also similar to that of their preceptors.

Interviews with the last two students’ FGDs and the last three preceptors did not yield new information (data saturation).^38^

All interviews were conducted in English, as it is the medium of instruction in all South African medical schools.^39^ An audio recorder was used to capture the discussions. On a daily basis, the research team kept record of reflective summaries for both the students and their preceptors to identify developing themes.^40^

### Data analysis

All recorded interviews were transcribed verbatim. An expert in linguistics checked each transcript to ensure accurate transcription and to minimise typographical errors. The principal researcher used both the inductive and deductive methods of data analysis.^41^ The inductive data analysis method was used for the unstructured broad exploratory question on the understanding of ‘generalist medical practice’ by both the students and their preceptors,^42,43^ while the deductive method was used for the preexistent semi-structured prompts,^44^ derived from the global attributes of ‘generalist medical practice’. Data elicited through the last prompt in the interview guide, which enquired about ‘any other comment’ by a participant regarding their understanding of ‘generalist medical practice’, were also analysed inductively. The inductive analysis was conducted following the method recommended by Bingam, whereby codes were identified, arranged into categories and finally into themes.^45^ Through deductive analysis, data were arranged according to the attributes of ‘generalist medical practice’ whereby the meaning of each attribute to the participants was recorded.^44^ The MAXQDA 2020 (Analytics Pro) software programme was used in the data analysis, which yielded 2179 data segments.

### Trustworthiness

As recommended for qualitative research studies,^46^ trustworthiness of this study was ensured by consideration of credibility, dependability, confirmability and transferability. To ensure credibility, a data set was created (Appendix 4), containing the study findings for participant validation regarding the accuracy of the information. Dependability, which relates to reproducibility of data, was ensured by providing a thick description of the study methods. Confirmability, which pertains to objectivity of the researcher in data collection and interpretation,^47^ was ensured by reflexivity, whereby the researcher was self-conscious of his influence on the participants as a trainer of medical students himself, allowing independent expression among all participants. For example, some students would clearly express their views that they did not think highly on generalist practitioner preceptors as they regarded them as lacking in knowledge and skills, compared to their specialist counterparts. The main researcher, himself a generalist practitioner, would acknowledge their viewpoint and never engage them in counterarguments. Transferability, the degree to which the study conclusions are applicable to other similar settings^46^ was ensured by providing sufficient description of the study setting and participants (thick description).^42^ Data triangulation was achieved through field notes taken during the interviews and reference to the students’ training manuals obtained from each medical school.^47^ Furthermore, the different participants (students and their preceptors) and methods used in data collection (FGDs and in-depth interviews) enhanced triangulation.

### Ethical considerations

Ethical clearance to conduct this study was obtained from the University of KwaZulu-Natal Humanities and Social Sciences Research Ethics Committee (No. HSS/2187/017D). This study was performed in line with the principles of the Declaration of Helsinki. Participation in the study carried no risk to the students. However, the study’s findings on the understanding of medical students and their trainers on ‘generalist medical practice’ could add an important dimension to the body of knowledge on the subject. Each consenting student and preceptor was requested to sign a written consent form for participation. Each participant was assured of the confidentiality of the information and that data storage for both electronic and hardcopy formats would be secured.

## Study’s findings

Students and their preceptors explained their understanding of generalist medical practice as: (1) the basic knowledge of medicine and in terms of the roles played by a practitioner in that position, (2) serving as the first point of patient contact with the healthcare system, (3) having knowledge of a wide range of conditions, (4) dealing with the undifferentiated patients, (5) involved in stabilising patients with emergency conditions who need referral, (6) providing continuity of care, (7) ensuring coordination of patient care, (8) providing holistic patient care, (9) advocating health promotion and disease prevention and (10) serving in primary health care (Table 2). The baseline characteristics of the students and their preceptors are available on Online Appendices 1 and Online Appendix 2 at the link provided under ‘Data availability’ below.

**TABLE 2:** Themes: students’ and preceptors’ understanding of generalist medical practice.

Themes	Subthemes
1. Basic knowledge of medicine	-
2. The point of first contact with patients	-
3. A wide range of conditions	General conditions
	Common conditions
	Minor conditions
4. The undifferentiated patient	-
5. Stabilises emergencies then refers	-
6. Continuity of care	-
7. Coordination of care	Ensuring patient advocacy
8. Holistic patient care	-
9. Health promotion and disease prevention	-
10. Operates at primary health care	-

### Basic knowledge of medicine

Students explained their understanding of generalist medical practice by describing the role of this care:

‘So, for me what I understand is just someone who has the ability to just be able to have a basic understanding at least in as many different conditions as possible. So, it’s just like, establish a sort of baseline for every condition out there at least, or every condition within your community at least ….’ (WTSM6.1, MBBCh 6, female student, 23 years)

They further elaborated: ‘So, for example when you rotate in orthopedics, you get the basic knowledge required of a generalist practitioner of how to manage basic orthopaedic cases’ (WSSM6.1, MBChB 6, 29 years). With basic knowledge of medicine, the generalist practitioners were further explained as ‘not specialised’ in any particular medical field, which was a limitation in their knowledge and skill:

‘Yes, my understanding is that they don’t have any specific specialty, uhm… they are not trained the way that leads them towards obstetrics or surgery or gynaecology or internal medicine so, they’re very limited in a way that they can treat or advise.’ (WSS6.6, MBChB 6, female student, 24 years)

In remaining on the basics required of generalist practitioners, one specialist preceptor admitted that his aim in the training of the UG medical students was not to turn them into ‘mini specialists’, but to give them basic medical education, taking into consideration that they were trainees who would exit their training with a generalist overview of medicine. He further highlighted the need for the training exposure to be in primary health care, rather than in specialist centres:

‘What I thought about is that, it works better if the specialties train[ed] the students to become generalists, if they understand the mandate to train a generalist and not a specialist. But, … the current model of spending time in gyne [*gynaecology*] for that long or in surgery for that long, … that speaks to that idea of a mini-specialist. Yes, maybe you should need to have exposure to it [*a specialty*], you need to see the stuff, but if they saw it in a primary health care environment … that would prepare them better.’ (BKZT7, clinical preceptor, Orthopeadics)

### The point of first contact with patients

Students asserted that generalist practice was the first point of patient contact with the healthcare system: ‘Generalist practice forms part of the first point of care which a patient would refer to when they’re having a problem’ (KZNS4.1, MBChB 4, male student, 21 years), and:

‘Uhm, my view of generalist practice is basically your first port of entry of the healthcare system … deals with the wide variety of problems … filters what is serious from the less serious. Those things that can be treated easily, I think that’s my view of generalist practice.’ (WTS6.3, MBBCh 6, male student, 25 years)

Students gave an account of the incremental nature of the complexity of conditions of the patients they encountered in primary health care settings, like clinics: ‘I think that’s very important in medicine, … we have that [*training*] occurring in different years, … more and more as you progress in your medical school career’ (KZSM6.2, MBChB 6, female student, 26 years).

The student preceptors were also aligned with this understanding: They’re [*generalist practitioners*] important as they are the first entry of any patient going into the health system’ (CSMT2, clinical preceptor, Psychiatry), and:

‘[…*W*]e make sure they know, specifically, on how to approach – they must first know how to identify the conditions and make sure they’re able to investigate and manage … they are the ones in first contact with the patient.’ (CSMT4, clinical preceptor, Obstetrics and Gynaecology)

### A wide range of conditions

As the first point of patient contact, the generalist practitioner was dealing with a wide range of medical conditions, which were described by students as general (not specialised), common and minor.

### General conditions

General conditions were explained as ‘anything that comes from the community …, any presentation from … any patient who comes in fresh from outside, even if they have their own chronic conditions’ (KZS5.3, MBChB 5, female student, 23 years). This called upon the generalist practitioner to possess an ‘all-disciplines’ competency:

‘So, it’s someone who possesses competence in all these fields [*of Medicine*] and is not just focused on a particular specialty. It’s someone who would be able to demonstrate a level of competence with whatever case that may come through his door.’ (KZS6.3, MBChB 6, female student, 26 years).

This scenario was thought to reflect the UG medical training in South Africa:

‘[*I*]n our setting in South Africa, doctors have been trained to fit anywhere within [*the*] specialties of medicine. For example, when you complete your internship, you can choose whether you want to be, maybe work in a gynae [*gynaecology*] or in orthopaedics, or anywhere.’ (SMS5.4, MBChB 5, female student, 23 years)

### Common conditions

The wide range of conditions also entailed common conditions: ‘My understanding … is, a generalist practitioner is basically a doctor who can function in any discipline and manage the common conditions that arise’ (KZS5.5, MBChB 5, male student, 24 years). There were various explanations on what qualifies a ‘common condition’, such as those indicated in students’ guidelines:

‘[… *T*]here are guidelines which are there, and they would tell you that this is diabetes, like in South Africa, this is how prevalent, let’s say diabetes is, this is how prevalent hypertension is.’ (WTS5.3, MBBCh 5, male student, 23 years)

And also, those conditions that were seen as prevalent in various communities, as determined by studies that had been conducted in those communities: ‘So, we would learn the conditions that are common in these communities’ (WTS5.3, MBBCh 5, male student, 23 years).

### Minor conditions

The wide range of conditions were further described as comprising ‘minor conditions’, which were understood as those that had low mortality and did not progress rapidly to kill a patient:

‘[*B*]y a minor basic problem [*it means*] it doesn’t lead to mortality in a fast rate per se. So, common cold has been well researched, and it’s been treated with success, it’s common and it doesn’t cause mortality in a fast rate. So, that, kind of, gives it the basis of “minor problem.”’ (KZS4.5, MBChB 4, female student, 26 years)

Students stated that, because generalist practitioners’ knowledge and skills were limited, they could only deal with minor uncomplicated conditions: ‘They [*generalist practitioners*] could be limited in skills, knowledge and experience with very complicated conditions that would usually be referred onward to the specialists’ (SMS6.1, MBChB 6, male student, 24 years). This limitation was viewed as potentially dangerous to a patient: ‘because sometimes generalists tend to ignore or mismanage certain conditions because they sort of lack the basic background’ (WSS5.4, MBChB 5, female student, 24 years). Students provided what they thought was the reason for the limitation – the limitation in resources at that level:

‘I think there are minor conditions that you [*as a generalist practitioner*] can manage with the resources that you have. But those that need further assessment, … need to be referred further.’ (WTS5.3, female student, 23 years)

Students’ preceptors also attested to the ‘broad base’ of common medical conditions manageable at a generalist level, which they understood to characterise generalist medical practice:

‘[*A*] generalist should be somebody who will be able to cope with the most common conditions present at whatever level of care. But, as I said, then you’d put a rider on and say, as a generalist you also need …, to recognise what is not of your level and refer it in time, properly.’ (CSMT6, clinical preceptor, Paediatrics)

And:

‘[*I*]t’s not [*that*] we are expecting them to know, you know, the canaries, it’ll be about assessing malnutrition, a child with HIV, a child with pneumonia, a child with diarrhoea, a child with common conditions, you know.’ (BKZT6, clinical preceptor, Paediatrics)

and ‘for each discipline, we make sure that the … common conditions are covered and [*at*] what scales we’ll be teaching the students’ (AWST5, clinical preceptor, Paediatrics).

#### The undifferentiated patient

At that first point of patient contact with the healthcare system, generalist practitioners deal with patients who have not been differentiated in terms of diagnosis:

‘My understanding of a general[*ist*] practitioner is that it’s a medical officer that sees undifferentiated patients like, whether surgical, medical or gynae [*gynaecological*] and then he, if need, can differentiate and refer them to specific specialties but then with minor ailments they manage, but with major, they refer to hospitals.’ (KZS4.3, MBChB 4, male student, 22 years)

The preceptors also described an undifferentiated patient in terms of a patient who has not yet been given a diagnosis. The ‘undifferentiatedness’ was one of the characteristics of patients at generalist level: ‘So when they are there [at generalist practice level] they are exposed to patients who are not yet diagnosed with any diseases, that is, undifferentiated patients’ (CSMT1, clinical preceptor, Family Physician); and:

‘[*T*]hey are encouraged to see that patient before anyone actually makes a plan on what needs to happen at that level, so that they see how the patient who comes [*with*] chronic cough comes to be called a pneumonia.’ (DWTT6, clinical preceptor, Paediatrics)

#### Stabilises emergencies

Students and their preceptors were of the understanding that a generalist practitioner is in possession of skills to recognise treatable conditions at his or her level, stabilise emergencies and refer them to appropriate specialties:

‘I think they should also be able to manage emergencies. Like, if a patient comes and maybe they have bled a lot and they have anaemia and they’re unconscious, they should be able to manage that patient until they’re stable enough to be referred.’ (KZS4.7, MBChB 4, male student, 22 years)

‘I think the generalist should be able to say, “how much of a threat to the person’s life is that condition,” and then look at … the resources that are available … the equipment … sometimes management might mean, stabilise the patient for referral.’ (KZS4.8, MBChB 4, female student, 23 years)

‘So, I think what this requires is … we need to define the competence of a generalist practitioner, if it is a Colle’s fracture, then you say, “You can do a Colle’s fracture, but this intra-articular disc fracture in a young person, is not a Colle’s fracture, this needs to go to the orthopod [*orthopaedic surgeon*].”’ (BKZT7, clinical preceptor, Orthopaedics)

#### Continuity of care

Students understood continuity of care to refer to forming a relationship with your patient as a medical practitioner:

‘So, it’s about firstly, I think, forming a relationship with your patient, being able to have a relationship with your patient, and also monitoring their conditions, being able to see when it gets worse, that’s what I think.’ (WTSM5.1, MBBCh 5, male student, 22 years)

and also about patient follow-up to check on the well-being of their patients:

‘But it’s all about following up, seeing your patients. Some doctors I think do have phones to actually check up on their patients, they also go to their patient’s house.’ (WTSM5.2, MBBCh 5, male student, 22 years)

Student preceptors also understood it as provision of a follow-up plan in patient care:

‘So, diarrhoea is a condition that I expect you [the student] to know everything about, a condition that I will expect you to do investigations, I will expect you to manage in the emergencies – fully, and I will also expect you to actually have a follow-up plan of chronic management if any, at that level.’ (DWTT6, clinical preceptor, Paediatrics)

The follow-up was at all levels:

‘[*I*]nteracting with patients and relatives at the community level, referring those that need to be referred to a hospital and when they are at the hospital, they meet those patients again.’ (AWST1, clinical preceptor, Family Medicine); and: ‘I do say to them [*medical students*], “if you’re looking after a patient and they go to the operating theatre, go with the patient so that you get to assist.”’ (DWTT5, clinical preceptor, General Surgery).

#### Coordination of care

Coordination of care was understood as referring to a multidisciplinary approach to patient care, which is facilitated by a generalist practitioner through mobilisation of all the team members:

‘So, I think a coordinated approach, means a multi-disciplinary approach, … it means that this generalist practitioner is able to function well within the medical team in order to achieve better patient outcomes and to sustain the management of that patient. So, basically it means that this generalist practitioner is able to work together [*with*] and mobilise all other members of the medical team in order to ensure that the best outcome is actually achieved for that patient.’ (SMSM5.1, MBChB 5, female medical, 22 years old)

The role of the generalist practitioner in referring a patient to appropriate professionals and overseeing that process was also mentioned:

‘Because generalists are the ones who are first contact of the patient, they are the ones who refer, they are the ones who know, who to do follow-ups on. They are like administrators. Engines of an organization.’ (SMS4.1, MBChB 4, female student, 22 years)

Student preceptors described it in terms of team leadership, whereby the generalist makes proper decisions on which clinician should be involved in the best interest of the patient: ‘Well, in my view it’s the doctor that should lead the team to offer coordinated healthcare services … [*to*] offer team-based care and leadership’ (DWTT1, clinical preceptor, Family Medicine):

‘So that’s bringing in the teamwork, it comes up in their training but there are certain conditions … even [*if*] you’re just a dermatologist you need to involve the other specialties.’ (AWST6, clinical preceptor, Dermatology)

### Ensuring patient advocacy

Students also mentioned the ‘advocacy for patients’ role of a generalist practitioner, which their preceptors did not mention. The students highlighted the advocacy role as ‘fighting’ for patients’ rights:

‘So, your role as a generalist in knowing what’s best for the patient, at times, when referring the patient, to fight for the patient because in most cases …, you call the tertiary hospital being X to refer the patient, and then the doctors there, … just pushes it off. So, if you just let it go like that, then your patient will end up dying. So, you have to advocate for the patient and make the doctor who is at the tertiary hospital understand.’ (WSS6.3, MBChB 6, male student, 24 years)

#### Holistic patient care

Students indicated that patient-centredness led to holistic patient care, which they also referred to as healthcare beyond disease symptoms: ‘So, he [*generalist practitioner*] approaches every sphere. So, you can see it as cultural, social, not only the disease or symptoms but everything’ (KZS6.6, MBChB 6, male student, 26 years). ‘I also think of it being where we manage patients more holistically, …’ (KZS4.2, MBChB 4, female students, 21 years). Students further understood it to span over specialist disciplines:

‘If it’s in paediatrics, it’s not just that the child has now got appendicitis, if the child is malnourished as well, you have to deal with all of that. If you need to see a dietitian, if you need to educate the parents, if you need to help with the grant because there are problems financially. You … have to deal with the whole patient.’ (WTS6.1, MBBCh 6, male student, 23 years)

The holistic patient care enabled the generalist practitioner to take into consideration the biopsychosocial aspects of the patient:

‘Oh, OK, so … when we treat a patient … we shouldn’t just treat the biological aspect only, but also we must take into account the social aspects, how the person lives from home, how they are uhm … basically all the social aspects and then also you must look at the psychosocial ones …’ (WTS4.1, MBBCh 4, male student, 23 years)

A psychiatrist added the spiritual dimension and described holistic patient care as addressing the bio-psycho-socio-spiritual aspects of the patient:

‘I mean if you look at the psychiatric diagnosis according to DSM-5 [*The Diagnostic and Statistical Manual of Mental Disorders, Fifth Edition*], … we train these students to do a dynamic formulation of the patient, looking at predisposing factors, … perpetuating factors, prognostic factors and then in their management strategy, particularly in regard to a bio-psycho-socio-spiritual context, how they would manage this patient.’ (DWTT2, clinical preceptor, Psychiatry)

It was further explained as putting the patient in the centre:

‘[*G*]eneralism is really putting the person in the centre, a holistic and ecological worldview, and it looks at relationships and connections, a lot more than categories.’ (BKZT1, clinical preceptor, Family Medicine)

#### Health promotion and disease prevention

Students and their preceptors understood health promotion and disease prevention as another role of a generalist practitioner, whereby she or he educates the communities on health matters:

‘[*W*]hen you go into the community, you might be expected to do some health promotion. Like, we’re expected to go to a school and talk to the children at the school about different conditions, whether it’s depression, whether it’s teenage pregnancy, to educate different members.’ (WTS6.2, MBBCh 6, male student, 23 years)

‘I think they [*generalist practitioners*] are important because their main role is not only treating but is also to encourage the public about preventative measures.’ (SMSM4.1, MBChB 4, male student, 21 years)

Student preceptors mentioned both primary and secondary disease prevention: ‘… if there’s an index case of TB then all the family members are screened’ (DWTT3, clinical preceptor, Internal Medicine); and ‘I would talk about pneumonia, … how you prevent pneumonia, … the vaccines and the role of immunisation and prevention of that condition’ (DWTT6, clinical preceptor, Paediatrics):

‘The next level will be secondary prevention … if this individual already has the diabetes, now they come because their sugar is poorly controlled, so some level of secondary prevention happens there, … that the kidney failure does not develop, the heart failure does not develop, the strokes do not develop.’ (CSMT3, clinical preceptor, Internal Medicine)

#### Operates at primary health care

Generalist practice was further understood as operational at primary health care settings: ‘And also, a general practitioner deals with everything but at primary [*health*] level. So, anyone can come to you and you have to treat them’ (WTS4.1, MBBCh 4, male student, 23 years). By primary care levels, students were referring to community levels: ‘When you speak about a general practitioner, essentially you see a doctor that is able to serve the community, which they are based in’ (WTS6.4, MBBCh 6, male student, 23 years).

A generalist medical practitioner was explained as someone who operates at primary health care level:

‘Okay, I think we do understand it that way, they’ll be somebody who is trained and being able to manage conditions that they will see in their primary health setting and the conditions that they will see will encompasses many specialties.’ (CSMT7, clinical preceptor, Orthopaedics)

## Discussion

This study has demonstrated that UG medical students and their preceptors had commonalities in their understanding of generalist medical practice, in keeping with the internationally accepted attributes relating to the first point of patient contact, the undifferentiated patient, continuity and coordination of care, holistic care, health promotion and disease prevention and its relevance for primary health care.

### The theoretical frameworks

The common understanding of the students and their preceptors on ‘generalist medical practice’ could be explained through Vygotsky’s social constructivist theory, given that the students came to the medical schools as ‘novices’ in the field of medical practice and gained the understanding through interaction with their ‘knowledgeable others’, the latter communicating these attributes to the students.

Lave and Wenger’s theory of ‘situated learning’ also gave insight into the understanding of the students and their preceptors regarding ‘generalist medical practice’.^24^ The ‘situated learning’ theory was the fulcrum around which the student learning occurred. Students learnt in the company of peers and preceptors and learnt their understanding of generalist medical practice in that situation. Through legitimate peripheral activities, the novices become acquainted with the tasks, vocabulary and organising principles of the community’s practitioners. Language (medical jargon and tradition) was the central part in the CoP.^24^ The theories have been referred to in the following paragraphs.

The researchers in this study searched for comparative literature on the subject of generalist medical practice among students and their preceptors. In high-income countries, generalist medical practice (also referred to as ‘generalism’) at UG medical training is discussed mainly with the view to prepare students to follow a postgraduate career in general practice (GP),^15,48^ different from the low- and middle-income countries (including South Africa)^49^ where postgraduate specialisation in general medical practice is not necessarily a prerequisite for general practice.^50,51^ Literature indicates that students perceived ‘generalism’ to be of lower prestige in the medical profession.^52,53,54^ These perceptions were already present at the very start of medical school and seemed to be reinforced during UG training.^15^ The researchers of this study could not find studies conducted on medical students’ and their preceptors’ understanding of ‘generalist medical practice’. The literature they found focused mainly on the involvement of general practitioners in UG medical training.^55,56^ Therefore, there was limited opportunity to draw international comparisons on the subject.

### Basic knowledge of medicine

The fact that students understood generalist medical practice as a limitation in the practice of medicine as they perceived it to only relate to basic and shallow knowledge of medicine needs to be put into its proper perspective. Through this basic medical knowledge, generalist medical practitioners cater for the needs of the majority in the community, and if implemented appropriately, can ensure efficiency of the healthcare systems in solving problems before they require specialised care as has been shown in literature.^57^ Therefore, rather than it being viewed as a limitation, it should be viewed as the strength of generalist medical practice.

The study’s finding that specialist preceptors indicated that they were not training students to turn them into ‘mini specialists’, but to give them basic medical education, was reassuring for generalist medical practice training. It should be encouraged in UG medical student training, as studies have shown that student preceptors themselves require training to be appropriate teachers.^58,59^

### First portal of entry

Students understood generalist medical practice as the first portal of entry of patients into the health system where patients are ‘filtered’. The filtering referred to patient screening for early detection of individuals with unrecognised diseases, as well as those with early stages of disease in the population.^34^ Student preceptors added that they guided students on the approach in dealing with these patients to enable them to identify a broad base of conditions spanning over many disciplines. Some of them may be medical emergencies needing stabilisation before referral to more specialised centres. This aspect of generalism is crucial in the training of medical students as it prompts them to think comprehensively,^35^ thus enhancing patient safety.^36^ In this way, student preceptors were taking the students through the ZPD to the level of expertise in patient care.^25^

### General medical conditions

Students further understood generalist medical practice as the field of medical practice dealing with ‘minor ailments’ which has been defined in literature as ‘conditions that will resolve on their own and can be reasonably self-diagnosed, for example, heartburn, nasal congestion, headache, etc.’^37^ However, some students also stated that they understood ‘minor ailments’ to be those that are ‘not complicated’. In this regard, their preceptors need to caution them, as there could be underlying complex conditions among those that may appear ‘minor’.^38^ To this effect, students need to be trained on the importance of knowing the natural history of an illness. This will entail the pattern of an illness that encompasses both its early and late presentations. This knowledge about the dynamic nature of illnesses over time will enable students to give appropriate safety-netting advice to their patients.^60^ A classic example would be a minor head injury where a patient is asked to look out for early symptoms and signs of worsening of the condition, such as drowsiness or inability to wake up.^61^ In such conditions, students need training to caution the patient and/or next-of-kin to report back immediately to the health facility when these occur for prompt intervention to be undertaken.

Furthermore, students and their trainers recognised the role of a generalist medical practitioner in managing emergency medical conditions. They provide the necessary treatment to those manageable within their scope of practice, stabilise and refer those who require specialised care. For the patient identified as requiring referral, evidence indicates that a generalist practitioner who has proper skills in patient referral, including transport arrangement and communication with the receiving hospital, prevents unnecessary delays, which would otherwise compromise patient care.^62^

### The undifferentiated patient

Both students and their preceptors understood the generalist medical practitioners to be dealing with the undifferentiated patient, whose condition needed proper assessment to determine treatability at generalist level or otherwise referral to the relevant specialties. The students’ preceptors leveraged that common understanding to guide students to implement their hitherto acquired clinical skills to work out for themselves a label (diagnosis) for the patient and to indicate their suggested management plan. There is evidence that UG medical students benefit from exposure and familiarisation with undifferentiated patients who are described as ‘patients walking through the door’, as these are the types of patients they will frequently encounter in generalist medical practice.^63^ Dealing with an undifferentiated patient provides students the opportunity to learn clinical reasoning, which entails synthesising the acquired information and skills to reach new conclusions about a patient’s condition.^25^ In this manner, Vygotsky’s ‘scaffolding’ principle becomes demonstrable, whereby students are taken through clinical conditions of gradual complexity.^21^

### Continuity and coordination of care

Regarding continuity of care, students understood that the generalist medical practitioner has the opportunity to establish a lifelong relationship with his or her patients from cradle to grave.^64^ Continuity of care challenged the students to be lifelong learners, given ‘the shifting ecology of the medical environment’.^65^ In keeping with their students, students’ preceptors understood continuity of care as relating to patient follow-up and building a continuing doctor–patient relationship with their patients. This addresses the important aspect of commitment to the patient as a person, rather than only their clinical conditions.^66^ Another attribute related to continuity of care is coordination of care, whereby a practitioner plays a key role in coordinating the team of healthcare professionals to potentiate patient care across diseases, settings and clinicians.^67^ The students were thus initiated to the concept of the ‘community of practice’ proposed by Wenger,^68^ enabling them to identify appropriate members of this community according to their role in the medical field. In coordinating patient care, the generalist medical practitioner ensures that patients’ right of access to available resources is ensured.^69,70,71,72^ In South Africa, commitment to coordination of care by generalist medical practitioners, including nurse practitioners, was found to be inadequate among generalists practicing in Cape Town in 2018.^73^ This is a call to ensure emphasis on coordination of patient care among all healthcare practitioners beginning at UG training level.

### Holistic patient care

Regarding holistic patient care, students understood it as taking into consideration factors beyond the clinical conditions that patients presented with, namely the psychosocial aspects. It is the duty of the generalist medical practitioner to adopt a holistic management approach, given the undifferentiated nature of the patients’ conditions they frequently encounter.^74^ Students’ preceptors described holistic patient care as the bio-psycho-socio-spiritual care. To this end, they ensured that student training did not only focus on the clinical conditions that patients presented with but also on the other patient aspects identified by the WHO in the definition of health, namely the mental and social aspects,^75^ which have also been described as the patient’s existential dimension.^9^

### Health promotion and disease prevention

The students and their preceptors understood ‘generalist medical practice’ to also entail health promotion and disease prevention. Literature indicates that these attributes link directly with the social determinants of health (SDH), such as water and sanitation, the inaccessibility of which frequently results in disease occurrence.^76^ This implies that health promotion and disease prevention cannot be adequately achieved without addressing these. In South Africa, the re-engineering of primary health care was introduced to increase access of health services to the general public, to address the SDH, thus improving the quality of health services in general.^77^ Nevertheless, the students’ and their preceptors’ understanding of interprofessional collaboration of various sectors to address the SDH fell outside the scope of this study, but should be considered in further studies.

### Relevance at primary health care

Students also understood the value brought by a generalist medical practitioner in primary health care, whereby the generalist’s competence encompasses a wide range of clinical disciplines and procedural skills requisite at primary health care in both chronic and emergency care, as also recorded in literature.^20^ This understanding could be leveraged on by the students’ preceptors in making the students understand the interconnectedness of generalist medical practice and primary health care.^48^ A practitioner serving in primary health care needs to understand the scope of the generalist medical practice. That understanding has been shown to set the scene in the delivery of comprehensive medical care.^78^

### Strengths and limitations

The study was conducted on only four of the nine medical schools in South Africa which limits transferability of the study findings. The variability of medical education among institutions necessitates caution in generalising the study’s findings to encompass the opinions of all medical students and their preceptors nationwide. Data collection occurred over a period of 5 years, which is the study limitation in terms of data consistency, as ideas of students and their preceptors could have developed and varied over this period. During the interviews, social desirability bias could have been introduced on the part of the medical students,^79^ whereby they could have behaved in a manner they thought would be acceptable to the principal researcher. The principal researcher was himself a preceptor. As far as the authors are aware, this is the first study conducted on the subject in South Africa.

### Study findings implications

The understanding of ‘generalist medical practice’ by medical students and their preceptors relating to the attributes discussed earlier was not dissimilar to the global understanding recorded in literature. However, the understanding that ‘generalist medical practice’ deals with uncomplicated medical conditions needs to be coupled with caution that ‘uncomplicated’ may turn out to have underlying complications. The understanding of ‘generalism’ as connoting ‘shallowness’ in medical knowledge and skills also needs correction. While it is true that ‘generalism’ deals with general (nonspecialised) medical conditions,^75^ emphasis should lie on training medical students to recognise medical conditions falling into the ‘generalist’ and ‘specialist’ categories to facilitate appropriate management – all in the interest of optimum patient care.

## Conclusion

The medical students and their preceptors in the South African medical schools demonstrated an understanding of the meaning of ‘generalist medical practice’ along internationally accepted attributes of the concept: the first point of patient contact, dealing with the undifferentiated patient, provision of continuity and coordination of care, holistic care, health promotion, disease prevention and relevance for primary health care. This common understanding should be reflected upon and leveraged on to produce a ‘basic medical practitioner’ at UG exit level. Nevertheless, questions remain for future studies to explore: Did the students and their trainers find the learning environment conducive for delivery of what they understood by ‘generalist medical practice’? Were there opportunities for collaborative engagement by the various disciplines on student training on the subject? It is recommended that further studies be conducted to address these pertinent matters in South African medical schools.

## References

[1] Brucker PC. A chance for the generalist? J Am Board Fam Pract. 1990;3(1 Suppl):15S–27S. 2363369

[2] Hashim MJ. A definition of family medicine and general practice. J Coll Physicians Surg Pak 2018;28(1):76–77. 10.29271/jcpsp.2018.01.76 29290201

[3] Mash R, Blitz J. Overcoming challenges in primary care education in South Africa. Educ Prim Care 2015;26(4):274–78. 10.1080/14739879.2015.11494355 26253068

[4] Heath I, Sweeney K. Medical generalists: Connecting the map and the territory. BMJ. 2005;331(7530):1462–1464. 10.1136/bmj.331.7530.1462 16356984 PMC1315659

[5] Kendrick T. 30th George Swift Lecture: Generalism in undergraduate medical education: What’s next? Br J Gen Pract. 2012;56(599):323–325. 10.3399/bjgp12X649296 PMC3361114 22687227

[6] Besigye I, Mash R, Essuman A, Flinkenflogel M. Conference report: Undergraduate family medicine and primary care training in sub-Saharan Africa: Reflections of the PRIMAFAMED network. Afr J Prim Health Care Fam Med 2017;9(1):a1351. 10.4102/phcfm.v9i1.1351 PMC5291078 28155289

[7] Mahoney S, Walters L, Ash J. Urban community-based medical education: General practice at the core of a new approach to teaching medical students. Aust Fam Physician. 2012;4:79–84. 23145410

[8] Derbyshire H, Rees E, Gay SP, McKinley RK. Undergraduate teaching in UK general practice. Br J Gen Pract. 2014;64(623):336–345. 10.3399/bjgp14X680113 PMC4032016 24868071

[9] Allen J, Gay B, Crebolder H & Heyrman J. The European definitions of the key features of the discipline of general practice: The role of the GP and core competencies. Br J Gen Pract. 2002;52:526–527. 12051237 PMC1314348

[10] Campbell J, Hobbs FDR, Irish B, et al. UK academic general practice and primary care Visible? Viable? Invaluable. BMJ. 2015;351:h4164. 10.1136/bmj.h4164 26231511

[11] Strasser R. Students learning medicine in general practice in Canada and Australia. Aust Fam Physician. 2016;45(1):22–25. 27051982

[12] McKinlay E, Pullon S. Having interprofessional education during the undergraduate years is essential for building teamwork skills in general practice: Yes. J Prim Health Care. 2014;6(4):331–333. 10.1071/HC14331 25485331

[13] Smith-Han K, Jaye C, FitzGerald R, Stein S. What do medical students learn about general practice in their undergraduate education? FoHPE [serial online]. 2012;14(3):84–96 [cited 2023 Oct 15]. Available from: https://search.informit.org/doi/10.3316/ielapa.562923250696356

[14] Royal College of General Practitioners. Medical generalism: Impact Report, 2013 [homepage on the Internet]. 2013 [cited 2021 Jan 19]. Available from: http://www.rcgp.org.uk/policy/rcgp-policy-areas/medicalgeneralism.aspx

[15] Fleming J, Patel P, Tristram S, Reeve J. The fall and rise of generalism: Perceptions of generalist practice amongst medical students. Educ Prim Care. 2017;28(4):250–251. 10.1080/14739879.2017.1306722 28372520

[16] Nutik M, Woods NN, Moaveni A, et al. Assessing undergraduate medical education through a generalist lens. Can Fam Physician. 2021;67(5):357–363. 10.46747/cfp.6705357 33980631 PMC8115970

[17] *Health Professions Act 56 of 1974*. Regulations relating to the registration of students, undergraduate curricula and professional examinations in Medicine” Published under Government Notice R139 in Government Gazette 31886 of 19 February 2009 [homepage on the Internet]. [cited 2023 Apr 24]. Available from: https://www.gov.za/sites/default/files/gcis_document/201409/31886139.pdf

[18] Reid SJ, Mash R, Downing RV, Moosa S. Perspectives on key principles of generalist medical practice in public service in sub-Saharan Africa: A qualitative study. BMC Fam Pract. 2011;2,67. 10.1186/1471-2296-12-67 PMC3142501 21726454

[19] Vygotsky LS. Mind in society: The development of higher psychological processes. Cambridge, MA: Harvard University Press; 1978.

[20] Drew C. Situated Learning Theory (Lave & Wegner) – Pros & Cons 2020 [homepage on the Internet]. [cited 2020 Apr 26]. Available from: https://helpfulprofessor.com/situated-learning-theory/

[21] Besar PH. Situated learning theory: The key to effective classroom teaching? IJECS. 2018;1(1):49–60.

[22] Nalliah S, Idris N. Applying the learning theories to medical education: A commentary. IeJSME. 20148(1): 50–57. 10.56026/imu.8.1.50

[23] Visser CL, Wouters A, Croiset G, Kusurkar RA. Scaffolding clinical reasoning of health care students: A qualitative exploration of clinicians’ Perceptions on an interprofessional obstetric ward. J Med Educ Curric Dev. 2020;7:2382120520907915. 10.1177/2382120520907915 32133416 PMC7040925

[24] Lave J, Wenger E. Situated learning: Legitimate peripheral participation. Cambridge: Cambridge University Press; 1991.

[25] Mabuza LH, Darong GG, Makhudu SJ, Drysdale RE, Moshabela M. The training of undergraduate medical students in general medical practice and primary health care: A scoping review. Open Public Health J. 2021;14:555–570. 10.2174/1874944502114010555.

[26] Hunter D, McCallum J, Howes D. Defining exploratory-descriptive qualitative (EDQ) research and considering its application to healthcare. J Nurs Healthc. 2019;4:2–8.

[27] Moshabela M, Moodley N, Campbell C, et al. Social accountability in the transformation of medical education to meet the needs of health care systems and local communities in South Africa [homepage on the Internet]. [cited 2023 Dec 20]. Available from: https://copsam.com/wp-content/uploads/2015/12/Sa-and-transformation-in-Medical-education.pdf

[28] Atkinson D. Rural-urban linkages: South Africa case study. Working Paper Series N° 125. Rimisp, Santiago, Chile: Working Group: Development with Territorial Cohesion. Territorial Cohesion for Development Program.; 2014.

[29] Persaud A, Bhugra D, Valsraj K, Bhavsar V. Understanding geopolitical determinants of health. Bull World Health Organ. 2021;99(2):166–168. 10.2471/BLT.20.254904 33551512 PMC7856359

[30] Golzar Jawad G, Omid T, Shagofah N. Convenience sampling [homepage on the Internet]. vol. 1, pp. 72–77. [cited 2023 Dec 15]. Available from: https://www.researchgate.net/publication/366390016_Convenience_Sampling/citation/download

[31] Ames H, Glenton Ct, Lewin S. Purposive sampling in a qualitative evidence synthesis: A worked example from a synthesis on parental perceptions of vaccination communication. BMC Med Res Methodol. 2019;19:26. 10.1186/s12874-019-0665-4 30704402 PMC6357413

[32] Kelly MA, Wicklum S, Hubinette M, Power L. The praxis of generalism in family medicine: Six concepts (6 Cs) to inform teaching. Can Fam Physician. 2021;67(10):786–788. 10.46747/cfp.6710786 34649904 PMC8516185

[33] Schaffir J, Morgan HK, Bhargava R, et al. To the point: Optimizing the learning environment in labor and delivery. Am J Obstet Gynecol. 2023;5(9):101090. 10.1016/j.ajogmf.2023.101090 37437693

[34] Bahari SF. Qualitative versus quantitative research strategies: Contrasting epistemological and ontological assumptions. Jurnal Teknologi. 2010;52:17–28.

[35] Roulston K. Reflective interviewing: A guide to theory & practice. Los Angeles, CA: SAGE; 2010, p. 116.

[36] Ritchie J, Lewis J, Elam G. Designing and selecting samples. In: Ritchie J, Lewis J, editors. Qualitative research practice: A guide for social science students and researchers. London: Sage, 2003; p. 77–108.

[37] Fern EF. The use of focus groups for idea generation: The effects of group size, acquaintance, and moderator on response quantity and quality. J Mark Res. 1982;19(1):1–13. 10.1177/002224378201900101

[38] Saunders B, Sim J, Kingstone T, et al. Saturation in qualitative research: Exploring its conceptualization and operationalization. Qual Quant. 2018;52(4):1893–1907. 10.1007/s11135017-0574-8 29937585 PMC5993836

[39] Hamad AA. Decolonization of medical education: A global screening of instructional languages and mother tongue dependence. J Med Surg Public Health. 2023;1:100007. 10.1016/j.glmedi.2023.100007

[40] Castleberry AN, Payakachat N, Ashby S, et al. Qualitative analysis of written reflections during a teaching certificate program. Am J Pharm Educ. 2016;80(1):10. 10.5688/ajpe80110 26941436 PMC4776288

[41] Azungah T. Qualitative research: Deductive and inductive approaches to data analysis. Qual Res J. 2018;18(4):383–400. 10.1108/QRJ-D-18-00035

[42] Shenton AK. Strategies for ensuring trustworthiness in qualitative research projects. Educ Inform. 2004;22(2):63–75. 10.3233/EFI-2004-22201

[43] Delve HL, Limpaecher A. Inductive content analysis & deductive content analysis in qualitative research (2023, March 10) [homepage on the Internet]. [cited 2023 Dec 2023]. Available from: https://delvetool.com/blog/inductive-content-analysis-deductive-content-analysis

[44] Pearse N. An illustration of deductive analysis in qualitative research. Rhodes University, Grahamstown, South Africa: In The 18th European Conference on Research Methodology for Business and Management Studies ECRM, Wits Business School, Johannesburg, South Africa, 20–21 June 2019 [homepage on the Internet]. [cited 2023 Sept 17]. Available from: https://eprints.lincoln.ac.uk/id/eprint/36421/1/ECRM19-Proceedings-Download.pdf#page=279

[45] Bingham AJ. From data management to actionable findings: A five-phase process of qualitative data analysis. Int J Qual Methods. 2023;22:16094069231183620. 10.1177/16094069231183620

[46] Anfara VAJ, Brown KM, Mangione TL. Qualitative analysis on stage: Making the research process more public. Educ Res. 2002;31(7):28–38. 10.3102/0013189X031007028

[47] Creswell JW. Educational research: Planning, conducting and evaluating quantitative and qualitative research. Upper Saddle River, NJ: Merrill Prentice Hall; 2012.

[48] The 2022 GP: A Vision of General Practice in the Future NHS. RCGP 2013 [homepage on the Internet]. [cited 2023 Dec 20]. Available from: https://www.srmc.scot.nhs.uk/wp-content/uploads/2021/07/The-2022-GP-A-Vision-for-General-Practice-in-the-Future-NHS-2013.pdf

[49] OECD Report [homepage on the Internet]. [cited 2023 Dec 29]. Available from: https://wellcome.org/grant-funding/guidance/low-and-middle-income-countries

[50] Matchaya M, Muula AS. Perceptions of medical students, faculty and private GPs towards the utilization of private GPs in the teaching of undergraduate medical students in Malawi: A qualitative study. Department of Community Health, University of Malawi, College of Medicine, Malawi (undated) [homepage on the Internet]. [cited 2023 Dec 20]. Available from: https://www.equinetafrica.org/sites/default/files/uploads/documents/MAThres.pdf

[51] Mohamoud G, Mash R. Clinical skills of general practitioners in Nairobi, Kenya: Across-sectional study. BJGP Open. 2022; DOI:10.3399/BJGPO.2021.0233. HW Carriers. General Medical Practitioner. How to be a General Medical Practitioner in South Africa [homepage on the Internet]. [cited 2023 Dec 20]. Available from: https://hw-careers.co.za/career/general-medical-practitioner/PMC9680751 35256356

[52] Pols DHJ, Kamps A, Runhaar J, et al. Medical students’ perception of general practice: A cross-sectional survey. BMC Med Educ. 2023;23:103. 10.1186/s12909-023-04064-z 36759816 PMC9912627

[53] Chellappan M, Garnham L. Medical students’ attitudes towards general practice and factors affecting career choice: A questionnaire study. London J Prim Care (Abingdon). 2014;6(6):117–123. 10.1080/17571472.2014.11494362 PMC4345787 25949732

[54] Koehler N, Mcmenamin C. Flexible but boring: Medical students’ perceptions of a career in general practice. Educ Prim Care. 2016;27:1–12. 10.1080/14739879.2016.1194360 27284679

[55] Grant A, Robling M. Introducing undergraduate medical teaching into general practice: An action research study. Med Teach. 2006;28(7):e19.2–7. 10.1080/01421590600825383 17594545

[56] Worley P, Silagy C, Prideaux D, Newble D, Jones A. The parallel rural community curriculum: An integrated clinical curriculum based in rural general practice. Med Educ. 2000;34(7):558–565. 10.1046/j.1365-2923.2000.00668.x 10886639

[57] Leinster S. Training medical practitioners: Which comes first, the generalist or the specialist? J R Soc Med. 2014;107(3):99–102. 10.1177/0141076813519438 24526462 PMC3938126

[58] Trainor A, Richards JB. Training medical educators to teach: Bridging the gap between perception and reality, Isr J Health Policy Res. 2021;10:75. 10.1186/s13584-021-00509-2 34915929 PMC8675462

[59] Ramanayake RP, De Silva AH, Perera DP, et al. Training medical students in general practice: A qualitative study among general practitioner trainers in Sri Lanka. J Fam Med Prim Care. 2015;4(2):168–173. 10.4103/2249-4863.154623 PMC4408694 25949960

[60] Silverston P. The safe clinical assessment: A patient safety focused approach to clinical assessment, Nurs Educ Today. 2014;34(2):214–217. 10.1016/j.nedt.2013.03.001 23541939

[61] Galgano M, Toshkezi G, Qiu X, Russell T, Chin L, Zhao LR. Traumatic brain injury: Current treatment strategies and future endeavors. Cell Transplant. 2017;26(7):1118–1130. 10.1177/0963689717714102 28933211 PMC5657730

[62] Ramanayake R, Ranasingha S, Lakmini S. Management of emergencies in general practice: Role of general practitioners. J Fam Med Primary Care. 2014;3:305–308. 10.4103/2249-4863.148089 PMC4311332 25657933

[63] Van Schalkwyk S, Blitz J, Couper I, et al. Breaking new ground: Lessons learnt from the development of Stellenbosch University’s Rural Clinical School. 2017 SAHR – 20 Year Anniversary Edition [homepage on the Internet]. [cited 2022 Feb 02]. Available from: https://journals.co.za/doi/pdf/10.10520/EJC-c80d2ad43

[64] Whitehead D. Before the cradle and beyond the grave: A lifespan/settings-based framework for health promotion. J Clin Nurs. 2011;20(15–16):2183–2194. 10.1111/j.1365-2702.2010.03674.x 21535458

[65] Priyadarsini S. Lifelong learning: The key to a successful medical career [homepage on the Internet]. [cited 2022 Nov 16]. Available from: https://www.docplexus.com/posts/lifelonglearningakeytosuccessfulmedicalcareer

[66] Asadi-Lari M, Tamburini M, Gray D. Patients’ needs, satisfaction, and health related quality of life: Towards a comprehensive model. Health Qual Life Outcomes. 2004;2:32. 10.1186/1477-7525-2-32 15225377 PMC471563

[67] Stille CJ, Jerant A, Bell D, Meltzer D, Elmore JG. Coordinating care across diseases, settings, and clinicians: A key role for the generalist in practice. Ann Intern Med. 2005;142(8):700–708. 10.7326/0003-4819-142-8-200504190-00038 15838089

[68] Wenger E. Communities of practice: Learning, meaning, and identity. Cambridge: Cambridge University Press; 1998.

[69] Nsiah C, Siakwa M, Ninnoni JP. Barriers to practicing patient advocacy in healthcare setting. Nurs Open. 2019;7(2):650–659. 10.1002/nop2.436 32089864 PMC7024610

[70] Vassbotn AD, Sjøvik H, Tjerbo T, Frich J, Spehar I. General practitioners’ perspectives on care coordination in primary health care: A qualitative study. Int J Care Coordinat. 2018;21(4):153–159. 10.1177/2053434518816792 PMC6297895 30595842

[71] Røsstad T, Garåsen H, Steinsbekk A, et al. Development of a patient-centred care pathway across healthcare providers: A qualitative study. BMC Health Serv Res. 2013;13:121. 10.1186/1472-6963-13-121 23547654 PMC3618199

[72] Spehar I, Sjøvik H, Karevold KI, et al. General practitioners’ views on leadership roles and challenges in primary health care: A qualitative study. Scand J Prim Health Care. 2017;35(1):105–110. 10.1080/02813432.2017.1288819 28277051 PMC5361414

[73] Christoffels R, Mash B. How well do public sector primary care providers function as medical generalists in Cape Town: A descriptive survey. BMC Fam Pract. 2018;19:122. 10.1186/s12875-018-0802-x 30025537 PMC6053747

[74] Nyangairi B, Couper ID, Sondzaba NO. Exposure to primary healthcare for medical students: Experiences of final-year medical students. S Afr Fam Pract J. 2010;52(5):467–470. 10.1080/20786204.2010.10874027

[75] Constitution of the World Health Organization. In: World Health Organization: Basic documents. 45th ed. Geneva: World Health Organization; 2005

[76] Braveman P, Gottlieb L. The Social determinants of health: It’s time to consider the causes of the causes. Public Health Rep. 2014;129(Suppl 2):19–31. 10.1177/00333549141291S206 PMC3863696 24385661

[77] Bheekie A, Bradley H. Re-engineering of South Africa’s primary health care system: Where is the pharmacist? S Afr Fam Pract. 2016;58(6):242–248. 10.1080/20786190.2016.1186365

[78] Stange KC. The generalist approach. Ann Fam Med. 2009;7(3):198–203. 10.1370/afm.1003 19433836 PMC2682975

[79] Bispo Júnior JP. Social desirability bias in qualitative health research. Rev Saude Publica. 2022;56:101. 10.11606/s1518-8787.2022056004164 36515303 PMC9749714

[80] Students’ & preceptors’ understanding of “generalist medical practice” [homepage on the Internet]. [cited n.d.]. Available from: https://figshare.com/s/948f50b84cb8af995381.

